# High-intensity interval training combined with cannabidiol supplementation improves cognitive impairment by regulating the expression of apolipoprotein E, presenilin-1, and glutamate proteins in a rat model of amyloid β-induced Alzheimer’s disease

**DOI:** 10.22038/ijbms.2024.79464.17210

**Published:** 2024

**Authors:** Mohammad Reza Kordi, Nooshin Khademi, Amir Mohammad Zobeydi, Samane Torabi, Esmaeil Mahmoodifar, Abbas Ali Gaeini, Siroos Choobineh, Parisa Pournemati

**Affiliations:** 1 Department of Exercise Physiology, Faculty of Sports Science and Health, University of Tehran, Tehran, Iran

**Keywords:** Alzheimer’s disease, Apolipoprotein E, Cannabidiol, Cognitive impairment, Glutamate, High-intensity interval – training, Presenilin-1

## Abstract

**Objective(s):**

Alzheimer’s disease (AD) is a major public concern and one of the primary types of dementia characterized by memory impairment and cognitive decline. Although the properties of exercise training and cannabidiol (CBD) treatments for improving AD have recently been revealed, the exact mechanisms remain unknown. Therefore, this study highlights the interactive impact of high-intensity interval training (HIIT) and CBD administration on improving cognitive impairment in a rat model of amyloid beta (Aꞵ)-induced AD through modulating the expression of apolipoprotein E (APOE), presenilin-1, and glutamate proteins.

**Materials and Methods:**

After acclimatization, the animals were randomly assigned into seven subgroups: control (CNT), Sham, Alzheimer (AL), Alzheimer + HIIT (AL + HIIT), Alzheimer + cannabidiol (AL + CBD), Alzheimer + CBD + HIIT (AL + CBD + HIIT), and model (sacrificed ten days after surgery to confirm the induction of AD) groups. To induce AD, rats received an intrahippocampal injection of Aꞵ. The animals in exercise groups performed the HIIT protocol, and the rats in CBD groups were administered 20 mg/kg CBD suspended in sesame oil for six weeks. Following the experimental protocol, serum and hippocampus tissue were collected for histopathological and western blot analysis.

**Results:**

Our findings indicated that both HIIT and CBD treatments were efficacious in ameliorating Aꞵ deposition and modulating biomarkers of AD, including APOE, presenilin-1, and glutamate. However, the interactive effect of HIIT and CBD supplementation was more effective.

**Conclusion:**

Our findings demonstrated the positive therapeutic effect of HIIT and CBD interventions, particularly HIIT combined with CBD, on alleviating AD.

## Introduction

Alzheimer’s disease (AD) is regarded as an irreversible, chronic neurodegenerative disease beginning with memory loss and likely resulting in communication problems, cognitive impairment, and poor judgment (1). AD is one of the leading models of dementia associated with reducing learning ability (2). It has been demonstrated that the formation of β-amyloid plaque (Aβ) from the amyloid precursor protein (APP) and hyperphosphorylation of Tau protein in the cerebral cortex and hippocampus are the leading causes of AD pathogenesis (3-5). To date, several studies have revealed that apolipoprotein E (APOE) is one of the major genetic risk factors that modulate Aꞵ clearance and accumulation in the hippocampus. APOE is a primary cholesterol carrier, crucial for improving brain injury and neuronal activity (6). In the brain, astrocytes and macrophages/microglia mainly generate APOE, and there are three types of APOE, APOE2, APOE3, and APOE4. APOE up-regulates APP transcription and Aꞵ production in an isoform-dependent way (ɛ4 > ɛ3 > ɛ2). Therefore, APOE is vital in Aꞵ accumulation, metabolism, and deposition (7-9). In addition, mutations in the presenilin-1 gene are related to an increased risk of familial AD (10). The mechanism by which presenilin-1 mutations lead to neurodegeneration and AD is still under debate. The amyloid beta hypothesis suggests that mutations in presenilin-1 account for AD pathogenesis via elevating Aꞵ production and apoptosis (11, 12). Presenilin-1 mutations increase APP processing and induce extreme production of Aꞵ42, which stimulates neurodegeneration and dementia in familial AD (10, 13-15). Available evidence suggests that presenilin-1 mutations have a pivotal role in hippocampal dysfunction and can directly result in apoptosis in the neurons (16, 17). Moreover, glutamate, an essential neurotransmitter in the brain and critical in memory formation, might be crucial to AD progression (18). Not only is Aꞵ production affected by glutamate, but also the levels of glutamate are altered by Aꞵ at the synapse. Thus, a minor alteration in the glutamate and Aβ concentrations could affect AD progression (18). Alteration in glutamate concentration depends on the AD stage. Multiple studies have shown a considerable decrease in glutamate concentration in the hippocampus, followed by Aꞵ deposition (19, 20).

Cannabidiol (CBD), an active agent of cannabis, has been gaining increasing attention due to its anti-oxidant and anti-inflammatory effects and its neuroprotective impact on decreasing neurotoxicity caused by Aꞵ and microglia in AD (21, 22). It has been demonstrated that CBD suppresses hippocampal neurodegeneration, decreases tau hyperphosphorylation, and also reduces Aꞵ aggregation (23-28). In addition to CBD, exercise training has been associated with improved cognitive function and brain structure in AD patients and older adults (29-31). High-intensity interval training (HIIT), which originates from athlete programs, has been shown to have a more significant effect on cardiovascular fitness than moderate-intensity continuous training (MCT) (32). HIIT has been found to reduce oxidative stress and apoptosis in the hippocampus of diabetic rats (33). Furthermore, HIIT has a lower time-wasting in clinical populations and may prove more enjoyable than MICT (34). Additionally, HIIT has a promising potential to ameliorate AD by regulating BDNF, oxidative stress, and inflammation and reducing Aꞵ accumulation (35-37). 

Regarding the previous studies, CBD and HIIT have positive advantages in alleviating AD progression. To date, published studies are limited regarding the effect of HIIT combined with CBD treatments on Aꞵ-induced AD, and the molecular mechanisms by which these interventions affect AD are not fully understood. Therefore, this study aims to investigate the role of HIIT and CBD supplementation in ameliorating cognitive impairment in a rat model of Aꞵ-induced AD via targeting APOE, presenilin-1, and glutamate. 

## Materials and Methods

***Animals***

Thirty-three Wistar rats (weighing 220–280 g, ten weeks of age, and male) were obtained from the Razi Vaccine and Serum Research Institute. The animals were maintained in cleaned cages (3 rats per cage) and in standard conditions, including 12-hr circadian rhythms, 22–25 °C temperature, 40–60% humidity, and free access to standard chow and water. All experiments were undertaken according to the Guide for Care and Use of Laboratory Animals (National Institutes of Health) (38). Moreover, all protocols in this research were approved by the ethics committee of the University of Tehran under the IR.SSRC.REC.1399.139 number (approval date: 2021/02/17).

***Experimental design***

**Figure 1:**
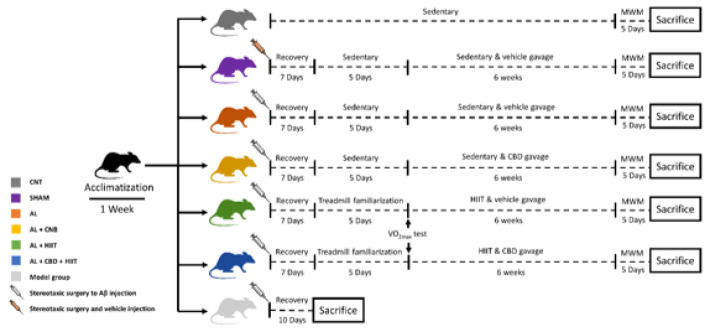
The schematic representation of the study design for the interventions and behavioral tests

In order to familiarize the animals with a new environment, they underwent an acclimatization phase for a week. Afterward, the rats were adapted to the rodent treadmill for five days at a speed of five to ten m/min and a time of five to ten min. Following the adaptation phase, the rats were allocated to six main groups (n = 5 per group), and one group (n = 3) to approve AD caused by Aꞵ was induced (Figure 1):

• Control group (CNT): The animals in this group remained sedentary (without any interventions).

• Sham group (Sham): 2.5 µl of the vehicle of Aꞵ (DMSO) was injected into the CA1 area of the hippocampus via stereotaxic surgery. They also received the vehicle (sesame oil) via gavage.

• Alzheimer group (AL): In this group, an intrahippocampal injection of Aꞵ42 was conducted via stereotaxic surgery, and the participants were sedentary.

• Alzheimer + HIIT (AL + HIIT): The animals in this group received an intrahippocampal injection of Aꞵ42. Afterward, they conducted high-intensity interval training and received a vehicle (sesame oil).

• Alzheimer + Cannabidiol (AL + CBD): In this group, AD was induced by stereotaxic surgery and administered with cannabidiol dissolved in sesame oil.

• Alzheimer + CBD + HIIT (AL + CBD + HIIT): The rats in this group received an intrahippocampal injection of Aꞵ42, performed HIIT, and were fed cannabidiol dissolved in sesame oil via gavage.

• Model group: To approve whether AD was induced, three rats were sacrificed ten days after Alzheimer’s induction via an intrahippocampal injection of Aꞵ42.

***Stereotaxic surgery and AD induction***

Before undertaking the surgery, the Aꞵ1-42 peptide (Sigma Aldrich; Cat number: SCP0038) was dissolved in a buffer solution of DMSO 3% (as a vehicle) at a concentration of 5 µg/1 µl. Afterward, the Aβ solution was incubated at 37 °C for seven days to induce fibril formation, which has neurotoxic effects (39). Following the preparation of Aꞵ1-42, the animals were anesthetized with intraperitoneal (IP) injections of ketamine (100 mg/kg) and xylazine (10 mg/kg) (40). Then, the animals were placed in the stereotaxic apparatus, and the CA1 region of the hippocampus was located based on the Paxinos atlas (coordinates 2.7 mm beneath the surface of the brain, 3.8 mm posterior to the bregma, and 2.2 mm lateral) (41). The injections were bilaterally conducted using a Hamilton syringe (2.5 µl each side) at a 1 µl/60 sec rate. Furthermore, the same surgery was performed on the animals in the sham group. However, they received the Aꞵ (DMSO) vehicle via bilateral intrahippocampal injection (2.5 µl). After stereotaxic surgery, the animals were allowed to recover for one week and monitored during recovery.

***CBD preparation and gavage***

Powdered cannabidiol (THC pharm, Germany) was suspended in sesame oil (Sigma Aldrich) for daily gavage at a dose of 20 mg/kg (42). Because of the insolubility of cannabidiol in water, sesame oil was used as a CBD solvent, increasing the bioavailability of cannabinoids (43). CBD was administered daily for six weeks through gavage, starting seven days following surgery. Other groups received sesame oil as a vehicle via gavage, except for the CNT group. 

***Exercise training protocol***

Seven days after the surgery, the animals in the AL + HIIT and AL + CBD + HIIT groups performed five days of treadmill adaptation on a rodent treadmill (Tajhiz Gostare Omide Iranian, Iran). Following the adaptation phase, the VO2max of the animals was measured based on the procedure used by Hoydal et al. (2007), demonstrating the relationship between running speed and VO2max (44). The HIIT protocol was implemented according to Naderi et al.’s research (2018) (45). In brief, the animals in the training groups were made to run five days per week (between 10 AM and 12 PM) for six weeks, once a day, and each session of training consisted of 30 min (Figure 1):

• Warm-up: The rats ran 6 min at 50-60% VO2max.

• Main training: The workload of the main body of training consisted of running at 90–100% VO2max (3 bouts, 4 min each) and 2 min of active rest at 50–60% VO2max between bouts. 

• Cool-down: Running for 6 min at 50-60% VO2max

In order to motivate the rats to run, mild electric shocks (about 0.5 mA) from a grid placed behind the treadmill were used.

***Morris water maze (MWM)***

Forty-eight hours after the last training session, MWM was conducted. MWM was performed in a black circular tank with a height of 50 cm, a diameter of 150 cm, and full of water (25 ± 2 °C). The tank was separated into four quadrants, including the first quadrant, second quadrant, third quadrant, and fourth quadrant. An escape platform with a diameter of 11 cm, a height of 30 cm, and a depth of 1 cm below the water surface was located in the middle of the fourth quadrant. Animals’ behavior and MWM’s parameters were documented using a video camera connected to the computerized ethovision Video Tracking System software (Noldus Co.). The test consisted of an acquisition phase and a retention phase, as follows:

• Acquisition trial: The animals were placed into the pool and allowed to swim and search the pool to discover the hidden platform for four trials per day (four days). They were allowed to explore the tank for 60 sec, and if they could not find the escape platform for 60 sec, they were guided to the escape/hidden platform by the researcher and remained on it for 20 sec. To assess the learning process, the time to reach (the mean value of four trials) the escape platform was recorded as escape latency (s).

• Probe trial: On day 5, the rats were subjected to the primary test, and the escape platform was eliminated. The animals were allowed to swim into the tank for 1 min, and the time spent in the platform’s location was recorded.

***Histological analysis***

*Thioflavin-S staining*

Thioflavin-S staining was applied to assess the Aꞵ depositions in the rats’ brains. Forty-eight hours after the experiments, the animals were sacrificed with CO2, and their hippocampus was perfused with 10% formalin for fixation (24 h). After that, the samples were embedded in paraffin blocks following the standard dehydration procedure. The paraffin blocks were cut with a microtome (5 µm thickness sections) and then mounted on microscope slides. In the staining procedures, the paraffin of hippocampus slides was removed with heat and xylene, followed by rehydration in descending ethanol (100%, 90%, 80%, 70%, and water, 1 min each solution). The hippocampus sections were treated with thioflavin-S for 5 min, then rinsed in 70% alcohol. Following washing by water, the green fluorescence of thioflavin‐S was detected using a fluorescence microscope. The percent of Aꞵ plaque formation was quantified using Image J software.

*Cresyl violet staining*

Cresyl violet staining was used to assess apoptosis in the experimental groups. Following tissue processing and deparaffinizing, hippocampus sample slides were stained with a cresyl solution (1%) for 5 min. Afterward, the slides were rewashed with water, restained in ascending ethanol solutions (just washing), and finally immersed in xylene. The percentage of apoptotic cells was quantified using Image J software.

***Western blot analysis***

After collecting hippocampus tissue samples, they were immediately placed in liquid nitrogen. Hippocampus samples were subsequently homogenized in ice-cold RIPA lysis buffer (CytoMatinGene, Cat No: CMGRIP50) supplemented with protease and phosphatase inhibitors. The total protein extract was collected from the supernatant after centrifuging the homogenized hippocampus samples at 12,000 rpm for 20 min at 4 °C. Equal amounts of protein samples (20–30 µg) were mixed with Laemmli buffer, denatured at 100 °C for 5 min, and loaded onto a polyacrylamide gel for SDS_PAGE separation. Electrophoresis was conducted at a constant voltage (100 V) for 1–2 hr until the protein bands reached the bottom of the gel. Proteins were transferred from the gel to a nitrocellulose membrane using a semi-dry transfer system. The nitrocellulose membranes were blocked with skim milk in Tris-buffered saline with 0.1% Tween-20 (TBST) (Sigma-Aldrich) for one hour at room temperature. Following blocking, primary antibodies targeting specific proteins were diluted in TBST with 1% BSA at a proper concentration, and the membrane was incubated overnight at 4 °C with gentle agitation. The membrane was washed thrice with TBST to remove excess primary antibodies. Afterward, it was incubated with HRP-conjugated secondary antibodies for two hours at room temperature. Protein bands were visualized through an enhanced chemiluminescent signal (ECL) substrate. The ECL signals were detected using X-ray film, and band intensities were quantified by Image J software. In order to detect the proteins, available antibodies were used, and the loading control was GAPDH.

***Statistical analysis***

All statistical analyses were conducted using GraphPad Prism 9 software, and *P*<0.05 was deemed statistically significant. Data are also reported as mean ± SD. One-way and two-way ANOVA followed by post hoc Tukey’s tests were used to analyze the data.

After 2 weeks of acclimatization, 33 male Wistar rats were randomly divided into six main groups (n = 5 per group), and a group (n = 3) to approve Aꞵ-induced AD induction. Afterward, the animals were subjected to main interventions for six weeks, followed by the MWM test. At the end point of the experiment, the hippocampus tissues were collected for histopathological and western blot analysis.

AL: Alzheimer’s group; CNT: Control group; HIIT: High-intensity interval training; CBD: Cannabidiol; MWM: Morris water maze

**Figure 2:**
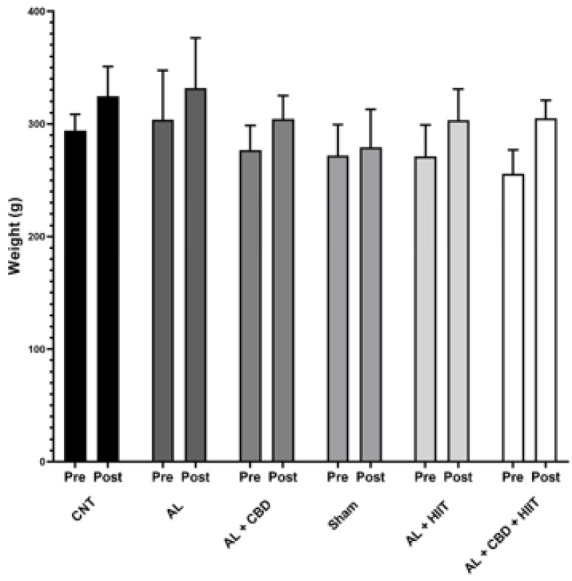
Average body weight changes of the male Wistar rats in the control and experimental groups

Data are reported as mean ± standard deviation (n = 5 per group). AL: Alzheimer’s group; CNT: Control group; HIIT: High-intensity interval training; CBD: Cannabidiol.

**Figure 3:**
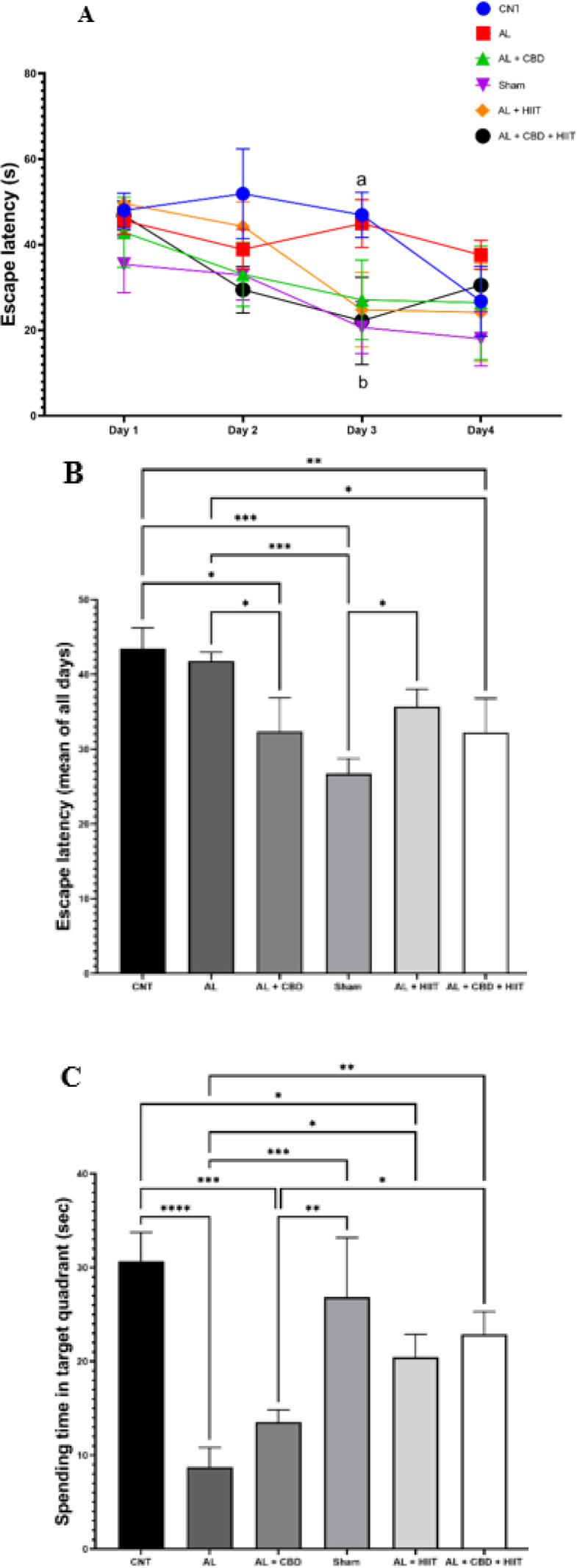
Impact of HIIT and CBD treatment on the Morris water maze test’s

A) escape latency (second) in the acquisition trial; B) escape latency (mean of all days); and C) spending time in the target quadrant in the probe trial

Values are reported as man ± sd (n = 3 per group). (a) statistically different from the Sham group (*P*<0.05) and (b) statistically different from the AL group. * *P*<0.05, ** *P*<0.01, *** *P*<0.001, and **** *P*<0.0001.

AL: Alzheimer’s group; CNT: Control group; HIIT: High-intensity interval training; CBD: Cannabidiol; MWM: Morris water maze

**Figure 4:**
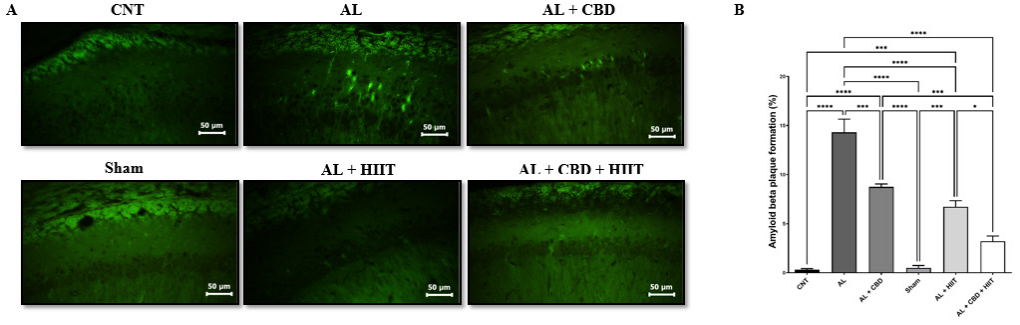
The effect of HIIT and CBD treatments on amyloid ꞵ (Aꞵ) plaque formation in the hippocampus of Aꞵ-induced rats

A) photomicrographs of the hippocampus following thioflavin-S staining (40 × magnification); B) quantitative data of the percentage of Aꞵ plaque formation in different groups. The values are presented as mean ± SD (n = 3 per group). * *P*<0.05, ** *P*<0.01, *** *P *<0.001, and **** *P*<0.0001.

AL: Alzheimer’s group; CNT: Control group; HIIT: High-intensity interval training; CBD: Cannabidiol

**Figure 5:**
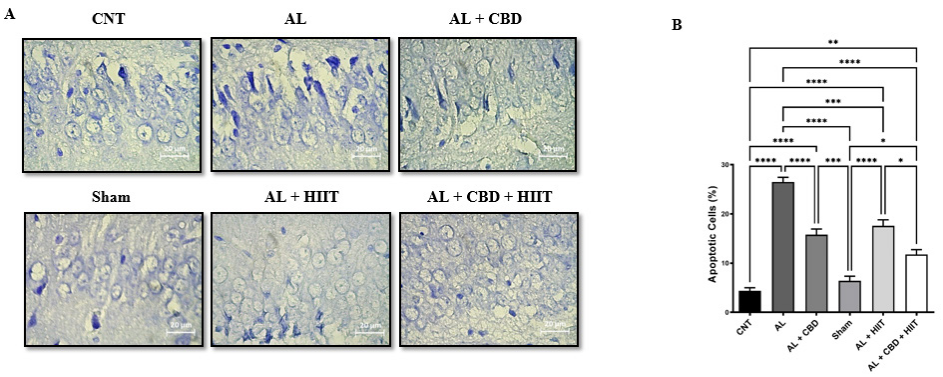
Evaluation of apoptosis in the hippocampus of Alzheimer’s rats

A) photomicrograph of hippocampus sections after cresyl violet staining (40 × magnification); B) the percentage of apoptotic cells quantified by Image J software. Data are presented as mean ± SEM (n = 3 per group). * *P*<0.05, ** *P*<0.01, *** *P*<0.001, and **** *P*<0.0001.

AL: Alzheimer’s group; CNT: Control group; HIIT: High-intensity interval training; CBD: Cannabidiol.

**Figure 6:**
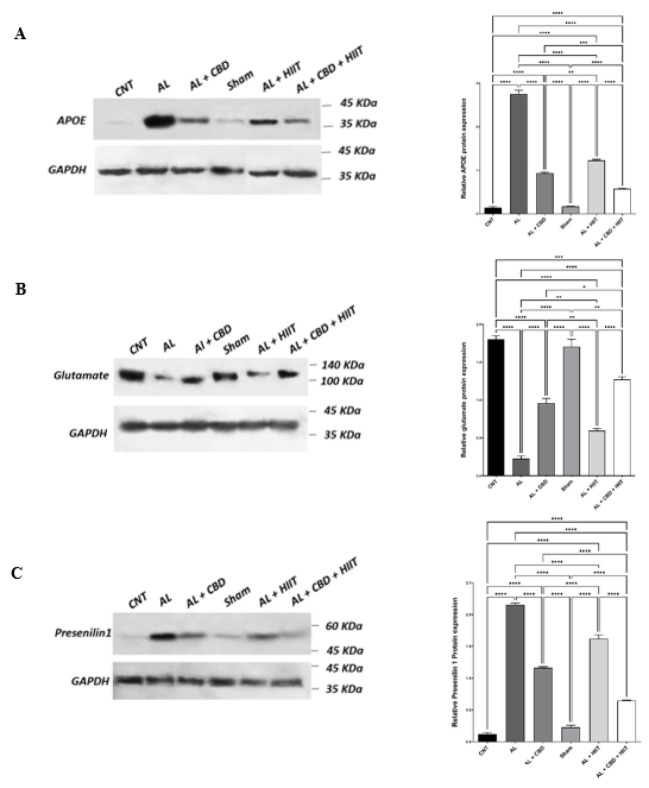
The impact of HIIT and CBD treatments on the expression of A) apolipoprotein E (APOE); B) glutamate; and C) presenilin-1 proteins

Values are presented as mean ± standard deviation (n = 3 per group). * *P*<0.05, ** *P*<0.01, *** *P*<0.001, and **** *P*<0.0001.

APOE: Apolipoprotein E; AL: Alzheimer’s group; CNT: Control group; HIIT: High-intensity interval training; CBD: Cannabidiol

## Results

***Confirming AD induction via sacrificing three rats***

The induction of AD was confirmed by sacrificing three rats ten days after amyloid beta injection. From the data in Figure S1A (supplementary data), the results of thioflavin-S staining in these rats (model group) established that AD was successfully induced, and then the main interventions were started. Furthermore, cresyl violet staining was performed to assess apoptosis in this group, and the results indicated high apoptosis in the hippocampus of these rats (Figure S1B).

***Evaluation of body weight***

At baseline, the rats’ body weight was identical in all groups. In addition, no significant differences were found between the last body weights of the rats from different groups. Moreover, a two-way ANOVA illustrated that HIIT and CBD supplementation did not significantly affect the animals’ body weight (Figure 2).

***Evaluation of memory acquisition (four days of MWM training)***

In order to evaluate memory acquisition, the escape latency to discover the hidden platform was recorded. According to the results of the two-way repeated measures analysis of variance obtained from four days of the acquisition phase, no significant differences in the escape latency to discover the escape platform were found between day one and day four in the AL group. However, the escape latency to discover the platform was significantly decreased from day one to day four in the AL + HIIT group and from day 1 to day 3 in the AL + CBD + HIIT group (*P*<0.05). Furthermore, on day 3, it was significantly higher in the AL group than in the AL + CBD + HIIT and Sham groups (*P*<0.05) (Figure 3A). As shown in Figure 3B, the mean analysis of all groups indicated that the mean of escape latency for all days was significantly higher in the AL group compared to the Sham group (*P*<0.01). Furthermore, it was significantly lower in the AL + CBD and AL + CBD + HIIT groups than in the AL group (*P*<0.05).

***Evaluation of spatial reference memory (probe trial)***

At the end of the acquisition trial (24 hr after the last intervention), on day 5, a probe test was performed to evaluate reference memory (the hidden platform was eliminated). A one-way ANOVA revealed that spending time in the target quadrant was significantly lower in the AL group than in the CNT group (*P*<0.0001). In addition, the AL + HIIT group significantly increased spending time in the target quadrant compared to the AL group (*P*<0.05), and it was significantly higher in the AL + CBD + HIIT group than in the AL group (*P*<0.01). Also, it was statistically higher in the AL + CBD + HIIT group compared to the AL + CBD group (*P*<0.05). However, no significant differences existed between the AL and AL + CBD groups’ spending time (Figure 3C).

***Determination of amyloid beta deposition***

To assess the formation of amyloid beta following AD induction, thioflavin-S staining was used, and the percent of amyloid beta deposition was quantified using Image J software (Figure 4). Aꞵ injection resulted in a significant increase in Aꞵ accumulation in the AL group compared to the CNT and Sham groups (*P*<0.01), and it was significantly higher in the AL group compared to other groups (*P*<0.05). Interestingly, HIIT and CBD treatments significantly diminished Aꞵ deposition compared to the AL group (*P*<0.01). However, Aꞵ accumulation was still significantly higher in the AL + HIIT and AL + CBD groups than in the CNT and Sham groups (*P*<0.01). In addition, Aꞵ deposition was statistically lower in the AL+ CBD + HIIT group than in the AL + HIIT (*P*<0.05) and AL + CBD groups (*P*<0.01), showing the synergic effect of HIIT and CBD treatments. There were no significant differences between Aꞵ plaque formation in the AL + CBD + HIIT group and the CNT or Sham groups.

***Cresyl violet staining***

Cresyl violet staining was carried out to evaluate apoptosis in the hippocampus of rats in different groups. It can be seen from the data in Figure 5 that there was a significant increase in apoptosis in the AL group compared to other groups (*P*<0.05). No significant differences were found between apoptosis of the CNT and Sham groups. It is apparent from this figure that HIIT and CBD treatments statistically decreased apoptosis in the AL + HIIT and AL + CBD groups compared to the AL group (*P*<0.05). However, apoptosis was significantly higher in the AL + HIIT, AL + CBD, and AL + CBD + HIIT groups than in the CNT and Sham groups (*P*<0.01). Also, the results of cresyl violet staining showed that apoptosis was statistically lower in the AL + CBD + HIIT group than in the AL + HIIT group (*P*<0.05). Conversely, no significant differences were discovered between the AL + CBD + HIIT and AL + CBD groups.

***Measuring hippocampal APOE protein expression in different groups***

A western blot analysis showed that the AL group illustrated a significantly higher APOE level than the CNT and Sham groups (*P*<0.01). Conversely, HIIT and CBD treatments statistically diminished the expression of APOE protein in the AL + HIIT, AL + CBD, and AL + CBD + HIIT groups compared to the AL group (*P*<0.01). The expression of APOE protein was significantly lower in the AL + CBD + HIIT group than in the AL + HIIT and AL + CBD groups (*P*<0.01). Furthermore, it was statistically lower in the AL + CBD group than in the AL + HIIT group (*P*<0.01). There were also no significant differences between the CNT and Sham groups, as demonstrated in Figure 6A. Therefore, both HIIT and CBD supplementation have a significant impact on reducing hippocampal expression of APOE. However, the combined effect of HIIT and CBD treatments on decreasing the expression of APOE protein was significantly higher than the impact of these interventions alone.

***Determination of glutamate protein expression in the rats’ hippocampus***

The results of one-way ANOVA regarding glutamate protein expression are shown in Figure 6B. From this figure, we can see that the expression level of glutamate was significantly lower in the AL group compared to other groups (*P*<0.01). Furthermore, HIIT and CBD treatments could successfully increase the glutamate expression level in the AL + HIIT, AL + CBD, and AL + CBD + HIIT groups compared to the AL group (*P*<0.01). However, it was still lower than the CNT and Sham groups (*P*<0.01). Moreover, HIIT combined with CBD supplementation significantly affected glutamate protein expression more than the AL + HIIT and AL + CBD groups (*P*<0.01). The expression level of glutamate was statistically higher in the AL + CBD group than in the AL + HIIT group (*P*<0.01). Therefore, CBD treatment was more effective despite HIIT’s efficacy in increasing glutamate in the hippocampus of AD rats. However, the synergic effect of these treatments was much more efficacious than CBD or HIIT interventions alone.

***Assessment of presenilin-1 protein expression in the hippocampus of rats***

The differences in the expression of presenilin-1 protein in different groups are highlighted in Figure 6C. The results indicate that the AL group displayed a significant increase in presenilin-1 expression compared to other groups (*P*<0.01). However, HIIT and CBD interventions reduced the expression of presenilin-1. Presenilin-1 expression was statistically lower in the AL + CBD, AL + HIIT, and AL + CBD + HIIT groups than in the AL group (*P*<0.01). Interestingly, HIIT combined with CBD administration was more effective in decreasing the expression of presenilin-1 compared to CBD or HIIT alone (*P*<0.01). Moreover, a significant difference was observed between presenilin-1 expression in the AL + CBD and AL + HIIT groups (*P*<0.01). Consequently, CBD may have a more significant effect on decreasing presenilin-1 in AD rats. However, the synergistic effect of HIIT and CBD supplementation can further reduce presenilin-1 expression.

## Discussion

The key finding of this study was that HIIT combined with CBD supplementation ameliorated AD induced by β-amyloid accumulation by regulating the expression of key biomarkers associated with AD, including presenilin-1, APOE, and glutamate proteins. Our findings confirmed that both treatments positively impacted the assessed parameters, which play a vital role in the context of cognitive decline and AD management. Our findings demonstrated that the combination of HIIT and CBD administration significantly improved cognitive impairment affected by AD, as evaluated through the Morris water maze test. These findings align with earlier research indicating exercise training and CBD’s potential to enhance cognitive abilities. For instance, Medhat and colleagues (2019) demonstrated that 30-minute four-week swimming training could improve cognitive impairment in rats suffering from AD (46). Other evidence, such as those conducted by Choi et al. (2014) and Rodrigues et al. (2010), suggests that exercise training could ameliorate cognitive dysfunction and enhance the brain function of AD rats (47, 48). Furthermore, many other studies show that HIIT has emerged as a powerful strategy for improving AD-induced cognitive impairment via anti-oxidative and neuroprotective properties (40). The possible mechanisms by which exercise training can improve cognitive deficit induced by AD are likely due to reducing oxidative stress, inflammatory response, and Aꞵ plaque in the brains of rats with AD (47). However, the current research findings do not support some of the previous studies. For example, it has been suggested that medium-intensity exercise has no significant impact on cognitive impairment caused by AD (49, 50). A possible explanation for this might be that training intensity was low in those studies, and intensity has a fundamental role in training adaptation. Regarding the impact of CBD administration on improving cognitive declines, recent studies are in line with our results, which indicated that chronic CBD supplementation had a mild impact on amelioration of cognitive dysfunction in a female AD mouse model (51). Moreover, Amini et al. (2021) confirmed that CBD treatment significantly improved memory, learning, and cognitive decline in a rat model of AD (52). These characteristics of CBD may be due to its therapeutic activities, including antioxidant, anti-inflammatory, and neuroprotective effects in a vast array of neurodegenerative diseases such as AD (21, 22, 42, 53).

A notable outcome of this paper is the observed reduction in Aꞵ accumulation in the hippocampus of the treatment groups. Aꞵ deposition is a hallmark of AD, and our findings confirmed that HIIT combined with CBD treatment might mitigate this pathological process. These results are in agreement with findings indicating that both HIIT and moderate-intensity continuous training (MICT) positively decreased Aꞵ deposition and ameliorated cognitive dysfunction induced by AD in APP/PS1 transgenic mice (54). Furthermore, Naderi et al. (2018) confirmed that HIIT could be a therapeutic tool for the clearance of Aꞵ deposition in the hippocampus of AD rats, equal to MICT (45). Several studies have documented the therapeutic properties of CBD in patients with neurodegenerative diseases, including epilepsy, AD, and multiple sclerosis. In this regard, Amini et al.’s (2021) findings are consistent with our data, showing that CBD treatment potentially decreased the accumulation of Aꞵ plaques and improved the condition of a rat model of AD (52). The theory behind these effects of HIIT and CBD treatments is still unknown and needs to be fully understood. Another important finding of our study was that the down-regulation of presenilin 1, a protein linked to the processing of amyloid beta, and the modulation of apolipoprotein E and glutamate protein expression, both associated with AD, point to potential mechanisms underlying the observed improvements. These findings warrant further investigation into the molecular pathways influenced by HIIT and CBD. The current study found that Aꞵ injection into the rats’ hippocampus led to a considerable increase in presenilin-1 and APOE expression and a decrease in the expression of glutamate protein. On the contrary, HIIT and CBD interventions significantly reduced the expression of presenilin-1 and APOE proteins and elevated glutamate levels. Interestingly, HIIT combined with CBD administration had a higher impact on the modulation of these proteins and the clearance of Aꞵ plaques. These results seem consistent with previous studies, which found that exercise training increased glutamate in the brain (55, 56). Our data is incompatible with Sarlak’s (2019) findings, demonstrating that aerobic training did not alter APOE mRNA expression. A possible explanation for this result is that our study’s training intensity was higher. Presenilin-1, which is associated with apoptosis in neurons and has a negative role in AD, is involved in Aꞵ deposition (16, 17). The decrease in apoptosis observed in the AL + HIIT, AL + CBD, and AL + CBD + HIIT groups is encouraging, as it suggests that the HIIT and CBD interventions may have a protective effect against neuronal cell death, an essential aspect of neurodegeneration. Several studies have indicated that exercise training and CBD supplementation protect against apoptosis in AD, and their findings align with our results (57-59).

The promising outcomes of this paper have significant clinical implications. A combined approach, including HIIT and CBD treatments, may hold promise for preventing or ameliorating cognitive impairment and AD progression. However, it is crucial to note that further research, including clinical trials with larger sample sizes and longer durations, is essential to confirm these findings and establish clear guidelines for implementation. We acknowledge several limitations, such as the relatively small sample size and the short duration of the interventions, which may impact the generalizability of our findings. In addition, we assessed limited AD markers and did not evaluate presenilin-1 receptors, glutamate receptors, or all types of APOE (ɛ4 > ɛ3 > ɛ2). Moreover, the exact mechanisms by which HIIT and CBD treatments impact the evaluated parameters require more in-depth investigation. There are still many unanswered questions regarding the precise molecular mechanisms by which HIIT and CBD interventions could alleviate AD. Further work is needed to establish the chronic impact of HIIT and CBD supplementation on AD caused by Aꞵ. Additionally, further research should be undertaken to investigate the safety and efficacy of CBD treatment in larger AD patients.

Therefore, the present study raises the possibility that HIIT combined with CBD administration can alleviate cognitive impairment by regulating the expression of APOE, presenilin-1, and glutamate proteins in a rat model of AD induced by ꞵ-amyloid.

## Conclusion

This study provides preliminary evidence that the combination of HIIT and CBD supplementation may positively affect cognitive dysfunction, Aꞵ accumulation, apoptosis, and the expression of key biomarkers associated with AD. Accordingly, the present study sheds new light on the emerging role of HIIT combined with CBD supplementation in improving AD progression via modulating the expression of APOE, presenilin-1, and glutamate proteins, reducing Aꞵ plaque, apoptosis, and decreasing cognitive impairment.

## Data Availability

The data supporting this study’s findings are available from the corresponding author upon reasonable request.
