# Supplementary material for: High-intensity interval training combined with cannabidiol supplementation improves cognitive impairment by regulating the expression of apolipoprotein E, presenilin-1, and glutamate proteins in a rat model of amyloid β-induced Alzheimer’s disease
Source: Iran J Basic Med Sci. 2024;27(12):1583–91. doi: 10.22038/ijbms.2024.79464.17210 (PMC11556759; doi:10.22038/ijbms.2024.79464.17210)
Supplement: Supplementary file 1 [file IJBMS-27-1583.S001.pdf]

A)

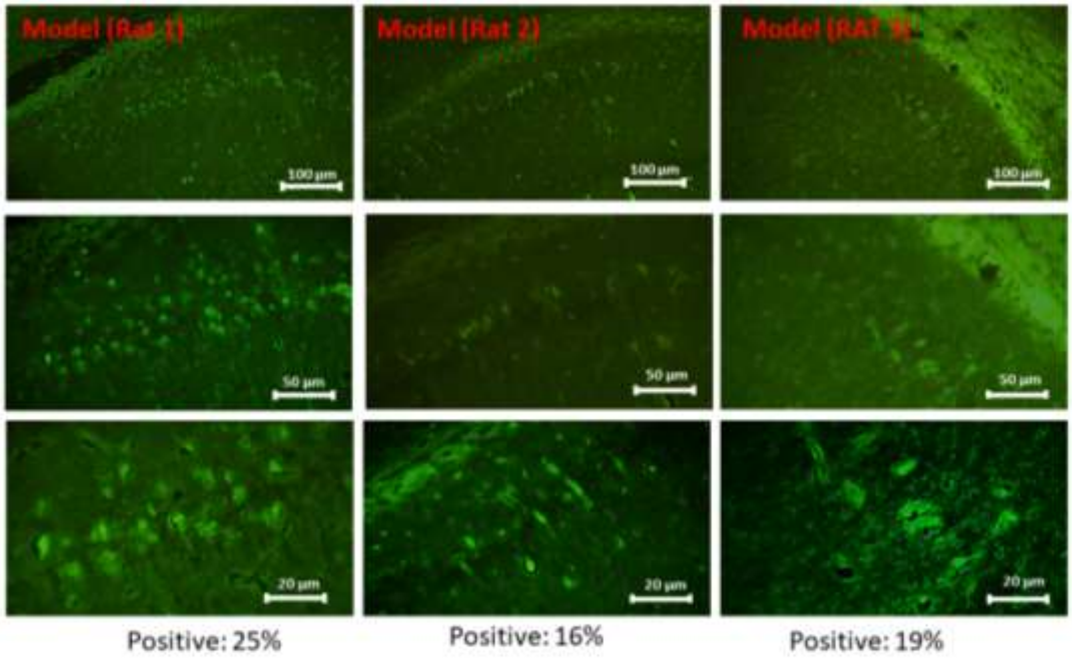

B)

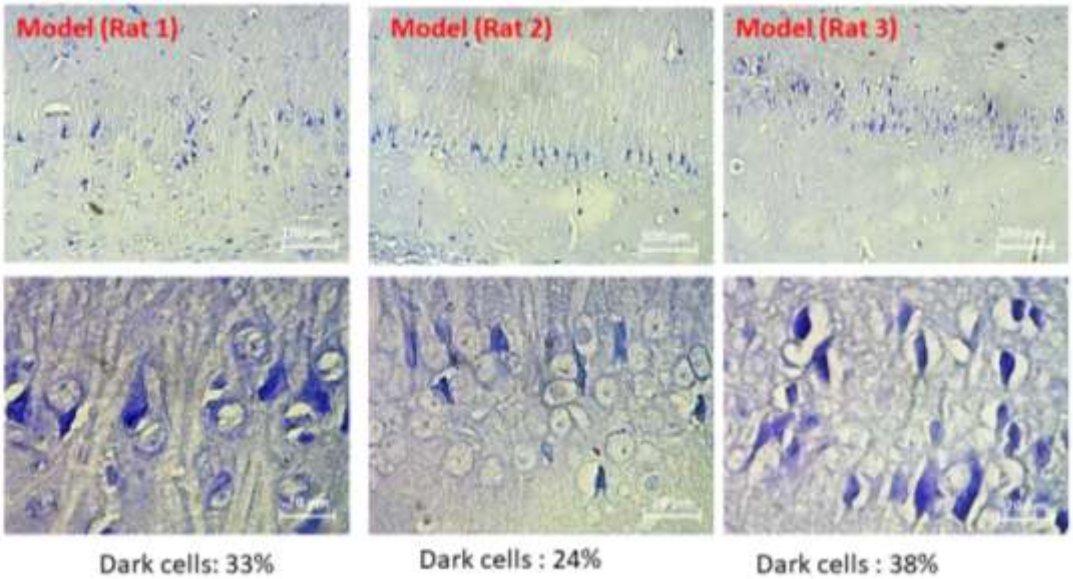

Figure S1. Thioflavin-S and cresyl violet staining in the model group. In order to confirm that Alzheimer's (AD) was induced, three rats were sacrificed ten days after β-amyloid (Aβ) injection. A) The percentage of Aβ accumulation in model group (40 × magnification) (B) the percentage of apoptosis caused by AD (40 × magnification).
